# 

**Published:** 1889-09

**Authors:** 


					﻿NOTICE.
All articles for publication, books for review, business communications, etc.,
must be sent to Editors, 1123.Spruce Street, Philadelphia. ;
Subscribers will please notice that this • Journal will be sent until arrears
are paid and it is ordered discontinued.
THE REPERTORY—CHAPTER II.
As we are nearing the completion of the chapter upon the
symptoms of the Head, ive find it will cover very nearly one
hundred printed pages; it would be very annoying to our
readers to have this given them in two numbers; we shall
therefore make zip a combined number for October and
November, issuing it early in the latter month.
This chapter upon the symptoms of the Head will be the
largest and the most complete of any ever issued and it is
hoped it will also be the most useful. The expense of print-
ing a repertory is very large, therefore we cannot afford to
mail this chapter to any subscriber who has not paid up his
subscription in full. This rule will be adhered to strictly.
				

